# Development of high-copy number plasmids in *Pseudoalteromonas haloplanktis* TAC125

**DOI:** 10.1007/s00253-023-12448-w

**Published:** 2023-03-13

**Authors:** Marzia Calvanese, Cecilia Balestra, Andrea Colarusso, Concetta Lauro, Christopher Riccardi, Marco Fondi, Ermenegilda Parrilli, Maria Luisa Tutino

**Affiliations:** 1 Department of Chemical Sciences, Federico II University of Naples, Complesso Universitario Monte S.- Angelo, Via Cintia, 80126 Naples, Italy; 2 Istituto Nazionale di Oceanografia e di Geofisica Sperimentale, Oceanography Division – OGS, Trieste, Italy; 3 Istituto Nazionale Biostrutture e Biosistemi I.N.B.B, Viale Medaglie d’Oro, 305-00136, Rome, Italy; 4 Department of Biology, Via Madonna del Piano 6, Sesto Fiorentino, 50018 Florence, Italy

**Keywords:** High-copy plasmid, *Pseudoalteromonas haloplanktis* TAC125, Recombinant protein production, Cold-adapted bacteria

## Abstract

**Abstract:**

The Antarctic bacterium *Pseudoalteromonas haloplanktis* TAC125 (*Ph*TAC125) is considered an interesting alternative host for the recombinant protein production, that can be explored when the conventional bacterial expression systems fail. Indeed, the manufacture of all the difficult-to-express proteins produced so far in this bacterial platform gave back soluble and active products. Despite these promising results, the low yield of recombinant protein production achieved is hampering the wider and industrial exploitation of this psychrophilic cell factory. All the expression plasmids developed so far in *Ph*TAC125 are based on the origin of replication of the endogenous pMtBL plasmid and are maintained at a very low copy number. In this work, we set up an experimental strategy to select mutated OriR sequences endowed with the ability to establish recombinant plasmids at higher multiplicity per cell. The solution to this major production bottleneck was achieved by the construction of a library of psychrophilic vectors, each containing a randomly mutated version of pMtBL OriR, and its screening by fluorescence-activated cell sorting (FACS). The selected clones allowed the identification of mutated OriR sequences effective in enhancing the plasmid copy number of approximately two orders of magnitude, and the production of the recombinant green fluorescent protein was increased up to twenty times approximately. Moreover, the molecular characterization of the different mutant OriR sequences allowed us to suggest some preliminary clues on the pMtBL replication mechanism that deserve to be further investigated in the future.

**Key points:**

*• Setup of an electroporation procedure for Pseudoalteromonas haloplanktis TAC125.*

*• Two order of magnitude improvement of OriR-derived psychrophilic expression systems.*

*• Almost twenty times enhancement in Green fluorescent protein production.*

**Supplementary Information:**

The online version contains supplementary material available at 10.1007/s00253-023-12448-w.

## Introduction

*Escherichia coli* and members of the genus *Bacillus* are the most commonly used bacterial cell factories for the production of recombinant proteins for different purposes, ranging from basic research to industrial or therapeutic applications (Westers et al. 2004; Tripathi and Shrivastava 2019). These organisms are the prokaryotic golden standards for several good reasons, including their well-known genetic setup, high growth rates in inexpensive media, and high-biomass yields (Mühlmann et al. 2017). However, these cell factories recurrently highlight significant product-related pitfalls, such as the formation of inclusion bodies, recombinant protein toxicity, and the lack of biological activity (Adrio and Demain 2014).

Different strategies have been pursued to overcome the observed limitations, for instance, by adjusting the growth conditions or by genetically engineering either the host strain or the design of the expression vectors (Francis and Page 2010). Unfortunately, despite the intensive application of the abovementioned interventions, a significant proportion of recombinant proteins (especially of human origin) remain unsuccessfully produced. In this respect, implementing new bacterial hosts as platforms for the production of difficult-to-express proteins is an attractive strategy. Although highly laborious, time-consuming, and expensive, this approach seems to be the only path to be pursued for the manufacture of high-value proteins, whose production in other bacterial hosts failed.

In this regard, cold-adapted bacteria have received considerable attention in the last two decades for their biotechnological potential as “cell factories.” *Pseudoalteromonas haloplanktis* TAC125 (*Ph*TAC125, recently renamed *P. translucida* TAC125) (Médigue et al. 2005) is the most intensively exploited psychrophilic bacterium for recombinant purposes. This Gram-negative strain, isolated from Antarctic seawater in 1992, was the first polar bacterium for which an efficient gene expression technology was developed (Parrilli et al. 2008), exploiting the autonomous replication sequence (OriR) derived from one of its endogenous plasmids, pMtBL (Tutino et al. 2001). The implementation of either constitutive or inducible promoters (Tutino et al. 2001; Duilio et al. 2004; Papa et al. 2007; Sannino et al. 2017; Colarusso et al. 2020), and the formulation of synthetic media based on the bacterial metabolism (Fondi et al. 2015; Sannino et al. 2017), allowed the production of several recombinant proteins (Vigentini et al. 2006; Dragosits et al. 2011; Unzueta et al. 2015a) at temperatures as low as − 2.5 °C (Sannino et al. 2017). These successful examples posed *Ph*TAC125 on the stage as one of the most promising alternatives to conventional cell factories for the successful manufacture of difficult-to-express proteins (Vigentini et al. 2006; Dragosits et al. 2011; Unzueta et al. 2015b; Calvanese et al. 2022).

Recently, an efficient isopropyl β-D-1-thiogalactopyranoside (IPTG)-inducible promoter was implemented in a pMtBL-derived psychrophilic vector, generating the pP79 vector (Colarusso et al. 2020). As the psychrophilic bacterium is devoid of any lactose import system, a genetically engineered bacterial strain was produced by genomically inserting of a functional copy of the lacY gene from *E. coli* into the lon-encoding gene of *Ph*TAC125 The resulting mutant (KrPL *LacY*^+^) has two desired features as an improved host for recombinant protein production, i.e., a faster inducer internalization rate, thanks to the mesophilic lactose permease and the absence of the Lon protease activity generally considered the major protease for the degradation of recombinant products (Colarusso et al. 2020).

Although the abovementioned approaches improved the efficiency of this unconventional cell factory, its industrial exploitation is still limited by the achievable average yields of recombinant products, which are often rather low (Colarusso et al. 2020). An experimental evaluation of the pMtBL copy number (PCN) revealed that at 15 °C the average PCN is 1.1 ± 0.09 in the mid-exponential phase, and 1.34 ± 0.06 in the late exponential phase (Qi et al. 2019). Even when the PCN is this low, pMtBL is extremely stable from generation to generation, thanks to the presence of a recently described partitioning system (Qi et al. 2019; Dziewit and Bartosik 2014). Given that all the *Ph*TAC125 expression plasmids developed so far are based on such replication origin, the recombinant production is likely limited by the low gene dosage achievable, regardless of the employed promoters and engineered strains. This observation raised our attention to pMtBL OriR and its replication efficiency making the development of psychrophilic replication origins endowed with higher copy number highly desirable and urgent. A rational approach toward this goal turned out unfeasible since the exact replication mechanism of pMtBL is still unknown, and the formulation of any hypothesis is made difficult by the lack of close relatives in the repository databases. Therefore, we decided to adopt a random approach, consisting in the construction of a library of psychrophilic plasmids, each containing a randomly mutated OriR. To obtain a fully representative number of independent recombinant psychrophilic clones an effective protocol for *Ph*TAC125 transformation was required. To fulfill this aim, we developed for the first time an electroporation procedure for the Antarctic bacterium transformation. The effect of each mutation on the average PCN was measured by the production of a fluorescent protein, upon IPTG induction of the culture. The psychrophilic cells were then sorted by fluorescence-activated cell sorting (FACS), and some cells with increased fluorescence were successfully isolated. Their molecular characterization allowed us to identify different mutant OriR sequences, all of which were able to establish the psychrophilic vector with a higher PCN than the wild-type one, reaching the enhancement of two orders of magnitude in the case of the mutant sequences we named Ori B40 and Ori Y18.

## Materials and methods

### Bacterial strains, media, and plasmids

The strains, plasmids, and oligonucleotides used in this study are listed in Table S1. *E. coli DH5α* strain was used for the cloning procedures. *E. coli* S17-1(*λpir*) was used as a donor in intergeneric conjugation experiments (Tutino et al. 2001). *E. coli* cells were routinely grown in the LB (10 g/L bacto-tryptone, 5 g/L yeast extract, 10 g/L NaCl) broth containing 100 μg/mL ampicillin (Sigma) if transformed at 37 °C. XL10-Gold ultracompetent cells were used for the transformation of the plasmid library after multi-site-directed mutagenesis. Transformation of XL10-Gold ultracompetent cells was optimized using NZY^+^ broth (10 g/L NZ amine (casein hydrolysate), 5 g/L yeast extract, and 5 g/L NaCl), complemented with 12.5 mL 1 M MgCl_2_, 12.5 mL 1 M MgSO_4_, 10 mL 2 M glucose, pH 7.5. KrPL, a cured *Ph*TAC125 strain, was used for protein expression and error-prone PCR (epPCR) library screening. The segregational stability assays and plasmid copy number (PCN) evaluation were also performed with the psychrophilic bacterium. KrPL was grown in the TYP broth (16 g/L bacto-tryptone, 16 g/L yeast extract, 10 g/L NaCl) during interspecific conjugations and precultures development. Expression experiments, plasmid stability, and PCN assays were carried out at 15 °C in the GG medium (Sannino et al. 2017) and 100 µg/mL ampicillin if transformed. Mutations of selected clones were confirmed via Sanger sequencing (Eurofins Genomics, Ebersberg, Germany).

### Plasmid vector preparation

The pMAI79-*R9-gfp* plasmid used for the library construction was developed from the pP79-*R9-gfp* vector (Colarusso et al. 2020)*.* Two unique restriction sites (*Not*I and *Asc*I) were introduced upstream and downstream of OriR into the original shuttle vector using the QuikChange Lightning Multi-Site-Directed Mutagenesis Kit (Agilent Technologies, Santa Clara, CA, USA) following the manufacturer’s instructions. Two mutagenic primers, Left OriR *Not*I Fw and Right OriR *Asc*I Rv (Table S1), were designed to introduce the two restriction sites. A small volume (1.5 μL) of the resulting PCR products was transformed into the XL10-Gold ultracompetent cells by chemical transformation. Then, the resulting plasmid, pMAI79-*R9-gfp*, was mobilized into KrPL by conjugation (Tutino et al. 2001). Selection of the recombinant transconjugants was performed at 15 °C in the presence of 50 µg/mL kanamycin and 100 µg/mL ampicillin.

### Construction of the plasmid library

A library of plasmids was constructed by introducing random mutations in OriR (842 bp) of the pMAI79-*R9-gfp* expression system using the GeneMorph II Random Mutagenesis Kit (Agilent Technologies, Santa Clara, CA, USA). To introduce mutations with low frequency (0–4.5 mutations/kb), 800 ng of target DNA was mutagenized by epPCR according to the manufacturer’s protocol using both primers mut_pP79-*R9-gfp*_Fw and mut_pP79-*R9-gfp*_Rv (Table S1). The epPCR reaction was performed in 50 μL containing 1 μL 2.5 U/μL Mutazyme II DNA polymerase, 2 μL 10 μM each primer, 1 μL 40 mM dNTP mix, 5 μL 10X Mutazyme II reaction buffer, and 4 μL distilled water. The obtained amplicons were subjected to *Not*I/*Asc*I double digestion and cloned into pMAI79-*R9-gfp*, which was previously digested with the same restriction enzymes. The plasmid library was transformed into the *E. coli DH5α* competent cells by chemical transformation. Immediately after transformation, the cells were diluted fivefold with LB medium, incubated for 3 h at 37 °C under 220 rpm shaking, and an aliquot of the cells (100 µL) was plated directly onto LB-agar plates to assess the complexity of the library. The remaining bacterial suspension was supplemented with glycerol at a final concentration of 15% (vol/vol), and 200 μL aliquots were frozen at − 80 °C. An aliquot of the cells was then thawed and spread with two dilutions (1/10 and 1/100) onto LB-agar supplemented with ampicillin. After 24 h at 37 °C, the colonies were counted. The library was large enough to generate at least 250,000 independent plasmid clones in *E. coli*.

### Electroporation of KrPL

The epPCR library was transformed into KrPL cells by electroporation. In this work, the electroporation protocol proposed by Kurusu and coworkers (Kurusu et al. 2001) was optimized for KrPL as follows. The strain was streaked from a glycerol stock (stored at − 80 °C) over a TYP agar plate that was incubated for 2 days at 15 °C. Then, one isolated colony was dispersed in 2 mL of the TYP and incubated overnight at 15 °C with 200 rpm agitation. Afterwards, the culture was diluted twice (1/100) in the same medium within the following 24 h. Finally, the last preculture was diluted in 50 mL of the TYP to start the growth at 15 °C until a value of 0.5 OD_600_ was reached. Then, the cells were recovered by centrifugation (15 min, 4000 rpm at 4 °C), and resuspended in 50 mL electroporation buffer (252 mM sucrose) (Kurusu et al. 2001). Cells were washed twice with smaller volumes of electroporation buffer and finally resuspended in 1 mL of the same buffer. For electroporation, 100 μL of electrocompetent KrPL cells were mixed with 1 μg of DNA and electroporated in 2 mm gap cuvettes (Gene Pulser/MicroPulser Electroporation Cuvettes, Bio-Rad Laboratories, Hercules, CA, USA) at 2.5 kV using a MicroPulser Electroporator (Bio-Rad Laboratories, Hercules, CA, USA). Cells were then immediately transferred to 900 μL TYP, incubated overnight at 15 °C with 200 rpm agitation, and then serial dilutions of the electroporated cell culture were plated out on the TYP-agar selective plates that were kept at 15 °C for 96 h to assess the complexity of the library. The remaining bacterial suspension was supplemented with glycerol at a final concentration of 15% (vol/vol), and frozen at − 80 °C. The number of transformants was estimated by counting the colonies on the plates and the total library size represented in the transformation was calculated (a library size of 10^5^ mutants approximately). A stock culture was used to inoculate 200 mL of the TYP supplemented with 100 µg/mL ampicillin and incubated overnight at 15 °C to complete library enrichment. When the culture medium became turbid (~ 2.5 OD_600_), it was supplemented with glycerol at a final concentration of 15% (volume/volume), and 5 mL aliquots were frozen at − 80 °C. Plasmid DNA was isolated from a small volume (4 mL) of the amplified library using a maxiprep DNA column (Qiagen, Hilden, Germany).

### FACS sorting

A stock culture (~ 10^5^ cells) of KrPL was slowly thawed on ice and inoculated into 50 mL of the TYP broth plus ampicillin (100 μg/mL) at 15 °C. After 24 h, the bacterial culture was diluted (100-fold dilution) in the GG medium-plus ampicillin (100 μg/mL) and kept overnight at 15 °C. After a series of serial dilutions in a crescent volume of the GG medium, the inoculum was grown in the GG medium-plus ampicillin (100 μg/mL). The expression was induced with 5 mM IPTG when the optical density of the sample ranged between 1 and 1.5. After 24 h from the induction, bacteria were analyzed and sorted using a Becton Dickinson Influx cell sorter (BD Biosciences, Franklin Lake, USA), at the cytometry laboratory of the Anton Dohrn Zoology Station, equipped with a 488 nm argon laser for excitation and 100 µm nozzle orifice. For maximum sorting purity, the “1 drop pure” sorting mode was used, ensuring the absence of non-target particles within the target cell droplet and the droplets immediately surrounding the cell. Accuracy was verified microscopically by examining the presence of fluorescent bacteria on a slide. The combination of Side Scatter (SSC) and Green fluorescence (530/40 nm) was used to detect and discriminate fluorescent-induced cells from not induced ones. Within the induced cells, three main populations were detected and only the one with the highest green fluorescence signal was sorted. A total of 200,000 fluorescent bacterial cells were selected, collected in a tube containing 500 µL of GG medium, and cultivated in 2 mL of the TYP-selective medium at 4 °C with agitation. After 5 days, the bacterial culture was transferred into the GG liquid medium at 15 °C and prepared for subsequent sorting under the same conditions described above. An aliquot of each sorted population was harvested during the exponential growth phase (OD_600_ = 1.0–1.5) in the GG medium, supplemented with 15% glycerol, and then stored at − 80 °C. Data acquisition and recording were achieved with the BD FACS Software software; graphs were drowned with FCS Express 6 Flow v 6.06.0025 (DeNovo Software, USA).

### Selection of mutants with higher plasmid copy number and fluorescence assays

Sorted cells were spread onto the TYP agar selective plates and incubated at 15 °C. After 96 h, individual clones were randomly selected and in a sterile 12-well flatbottomed polystyrene plate filled with 1.5 mL TYP medium under selective growth conditions. Then, the cells were transferred to the GG medium with a starting OD_600_ of 0.1 and induced into the exponential phase (OD_600_ = 1.0–1.5) with 5 mM IPTG. To determine the fluorescence intensity of R9-GFP, 2 OD_600_ of liquid cultures were centrifuged at 13,000 rpm for 10 min at 4 °C and the pellets were resuspended in 0.5 mL PBS. The fluorescence was measured (488-nm excitation—slit 3509-nm emission—slit 6) using a JASCO FP-750 spectrofluorometer at 25 °C with a 1 cm path length. Fluorescence was normalized by dividing the measured fluorescence intensity for appropriate dilutions of the cell suspensions. The data were processed using the Origin 81 software.

### SDS-PAGE

Recombinant protein production was also monitored by SDS-PAGE. Cell pellets (1 OD_600_) were harvested after 24 h from the induction and collected by centrifugation (15 min, 12,000 rpm, 4 °C). The pellets were solubilized in 60 µL of Laemmli buffer 4 × and boiled at 95 °C for 20 min and 1.5 µL of samples were analyzed with 12.5% polyacrylamide gel (SDS-PAGE). After the electrophoresis, the gels were stained with Coomassie brilliant blue dye for 2 h and destained with distilled water.

### Plasmid copy number determination via qPCR

Relative plasmid copy number (PCN) was calculated by the quantitative PCR (qPCR) method. Amplification and analysis were performed with a StepOne Real-time PCR System (Applied Biosystems, Foster City, CA, USA) and the SYBR® Green PCR Kit (Applied Biosystems, Foster City, CA, USA). Particularly, the *PSHA_RS10135* gene was always used to detect the chromosome in the samples. The *R9-gfp* gene was used for the detection of the plasmid. Each couple of primers (Table S1) was selected using the free Primer 3 web software. For the PCN estimation, total DNA was extracted from 1 OD_600_ pellet collected at 8 h from induction using the Bacterial DNA kit (D3350-02, E.Z.N.A™, OMEGA bio-tek, Norcross, GA, USA) following the manufacturer’s instructions. DNA concentrations were measured with a NanoDrop TM 1000 Sp spectrophotometer (Thermo Fisher Scientific, Waltham, MA, USA) at the absorption of 260 nm. The ratios of A260/280 and A260/230 were calculated to estimate the purity of the extracted DNA. The integrity of the extracted DNA was assessed by gel electrophoresis and visualized using the ChemiDoc MP Imaging System (Bio-Rad Laboratories, Hercules, CA, USA). PCR reactions were prepared in 10 µL mixtures containing 1 × PowerUp SYBR Green Master Mix (Applied Biosystems, Foster City, CA, USA) with ROX as passive reference dye and Uracil-DNA glycosidase (UDG) to eliminate contaminations, 400 nM of each primer and 1 µL of sample and the reaction master mixes were aliquoted in three wells of a reaction plate. Finally, the plate was sealed with an adhesive cover (Applied Biosystems, Foster City, CA, USA). The thermal cycling protocol was as follows: UDG activation for 2 min at 50 °C; initial denaturation for 10 min at 95 °C; 40 cycles of denaturation for 15 s at 95 °C alternated with annealing/extension steps for 1 min at 60 °C. Each reaction was performed in triplicate. Cycle threshold (Ct) values were determined after automatic adjustment of the baseline and manual adjustment of the fluorescence threshold using the LightCycler® 96 Software. Standard curves were generated using tenfold serial dilutions of either *R9-gfp* or *PSHAa2051* genes. The amplification efficiency (E) of each gene was calculated from the slope of the relative standard curve (E = 10^(−1/slope)^). The PCN was determined considering Ct values for the two amplicons (chromosome-c and plasmid-p) and the amplification efficiency of the plasmid (E_p_) and chromosomal gene (E_c_) with the following equation: PCN = (E_c_)^Ctc^/(E_p_)^Ctp^ (Škulj et al. 2008).

### Plasmid loss frequency assays

The plasmid segregational stability of the progenitor and some mutants was investigated under non-selective growth conditions. A single colony of each strain was collected from TYP agar selective plates and inoculated in the TYP plus antibiotic at 15 °C under agitation. Then, the cells were diluted to 0.1 OD_600_ in the GG medium containing the selective agent. After 24 h of growth at the same temperature, the cultures were 1/20 diluted daily in antibiotic-free GG to keep them constantly in the exponential phase (0.2–1.5 OD_600_). At precise intervals of time, the cultured cells were diluted by a factor of 10^4^ and spread onto antibiotic-free-TYP agar plates. After 2 days of incubation at 15 °C, at least 30 colonies were selected and plated onto the TYP agar with and without ampicillin and incubated at 15 °C for 2 days. The plasmid stability was determined until 80 generations using duplicates. The maintenance of each plasmid in the KrPL bacterium was calculated by the number of colonies grown on the TYP-agar plates with ampicillin divided by the number of colonies grown on the TYP-agar plates without ampicillin.

### Sequence analysis

Detection of potential open reading frames (ORFs) was performed with the ORFfinder software at NCBI (Wheeler et al. 2003). The standard genetic code and “atg, gtg, ttg, ctg” as alternative start codons were used. The minimum ORF size was restricted to 30 amino acids. Multiple sequence alignments were conducted in Clustal Omega – EMBL. The predicted secondary structure of DNA and RNA molecules was performed by the Mfold web server (Zuker 2003).

### Deletions of DNA sequences in the replication origin

The PCR-mediated plasmid DNA deletion method was used to delete DNA sequences in OriR of pMtBL-derived plasmids. Primers were designed to amplify the entire circular sequence of the plasmid except for the specific region that was to be deleted. The 5′ ends of the primers include the cutting sequence of the chosen restriction enzyme. The restriction sites, *Nsi*I and *Bsi*WI, were chosen to remove the putative secondary structures, named STEM-LOOP I and STEM-LOOP II. The deletion of the fragment of 342 bp (from 1918 to 2259 bp) was performed using the *Not*I restriction site. The primers used for deletions are listed in Table S1. The PCR reactions were carried out using the Phusion® High-Fidelity DNA Polymerase (Thermo Fisher Scientific, Waltham, MA, USA) and the pMAI79-*R9-gfp* plasmid as the template. The PCR products were then digested with the chosen restriction enzyme (described above) and individually cloned into pMAI79-*R9-gfp* previously digested with the same restriction enzymes. The three mutated vectors were mobilized into KrPL by intergeneric conjugation (Tutino et al. 2001).

### RNA isolation and RNA-Seq data analysis of pMtBL

For the RNA-Seq experiment, the *Ph*TAC125 bacterium was grown in the GG medium at 15 °C, and 1 OD_600_ pellets were collected during the exponential growth phase (~ 1/1.5 OD/mL) by centrifugation (10 min, 13,000 rpm, 4 °C), washed in RNAse-free PBS three times and stored at – 80 °C. Biological triplicates were performed.

Total RNA was extracted using the Direct-zol RNA Kit (Zymo Research, Irvine, CA, USA) following the manufacturer’s instructions. Contaminating genomic DNA was then removed through treatment with RNAse-free DNase I (Roche, Mannheim, Germany). The sequencing service was provided by the Genome Research Center for Health (Campus of Medicine of the University of Salerno, Baronissi, Italy). Afterwards, all samples were analyzed and assessed for base call quality and adapter content using fastp (Chen et al. 2018) version 0.22.0, allowing down to a mean quality threshold of 20 (i.e., probability of incorrect base call of 1 in 100) and minimum read length of 40 nucleotides. A median of 98.9% of the reads across all samples passed the quality check, indicating that sequencing was carried out pristinely, and our data were biologically reliable. An index file for reads alignment was produced through Bowtie2 (Langmead and Salzberg 2012) version 2.2.9 using the entire genomic sequence of *Ph*TAC125 (NCBI Reference Sequence ASM2608v1) with the additional concatenation of its recently discovered plasmids, pMEGA (NZ_MN400773.1) and pMtBL (NZ_AJ224742.1). Reads mapping (also with Bowtie2, in very sensitive mode) produced a 99.98% alignment rate. Samtools version 1.11 (Li et al. 2009) with flags –f 99/147 and –f 83/163, respectively, were used to partition sequencing reads that mapped to the forward and reverse strand. After reads partitioning, the same suite was utilized to calculate per-nucleotide depth. This stage of the analysis confirmed a forward-stranded protocol (first strand library, data not shown), and allowed to draw a strand-sensitive profile of sequencing depth (Fig. S4). All command line software was run in multithreading on at least one AMD Opteron Processor 6380, 2.5 GHz.

## Results

### Set up of a library of psychrophilic plasmids containing randomly mutated pMtBL replication origins

The preparation of the plasmid library containing the randomly mutated OriR started with the construction of pMAI79-*R9-gfp*, a direct derivative of the IPTG-inducible plasmid pP79-*R9-gfp* (Colarusso et al. 2020), which harbors the *R9-gfp* gene under the control of the *Ph*TAE79 *lacR-lacZ* regulatory elements (Colarusso et al. 2020) (Fig. S1). The pMAI79-*R9-gfp* plasmid differs from its ancestor as two restriction sites (*Not*I and *Asc*I, respectively) were added by site-directed mutagenesis upstream and downstream of the pMtBL OriR sequence (corresponding to the sequence from nucleotide 1918 to 2759 of the GenBank database entry NZ_AJ224742.1) (Tutino et al. 2001).

As for the OriR sequence random mutagenesis, the psychrophilic origin of replication was amplified by *error-prone* PCR with low mutation frequency, suitably digested by *Not*I and *Asc*I, and then ligated into pMAI79-*R9-gfp* corresponding sites. The library was first transformed into *E. coli* cells, and then a fully representative number of independent plasmids were extracted and used to transform KrPL cells, a *Ph*TAC125 strain devoid of the endogenous pMtBL plasmid (Colarusso et al. 2020), with an electroporation procedure suitably developed for the Antarctic bacterium. The strategy to achieve an effective electroporation was set up by modifying Kurusu’s protocol (Kurusu et al. 2001). In particular, buffer composition and proper voltage settings were found to be key elements for successful electroporation of Antarctic cells.

### FACS sorting of psychrophilic cells harboring the randomly mutated plasmid library

**Fig. 1:**
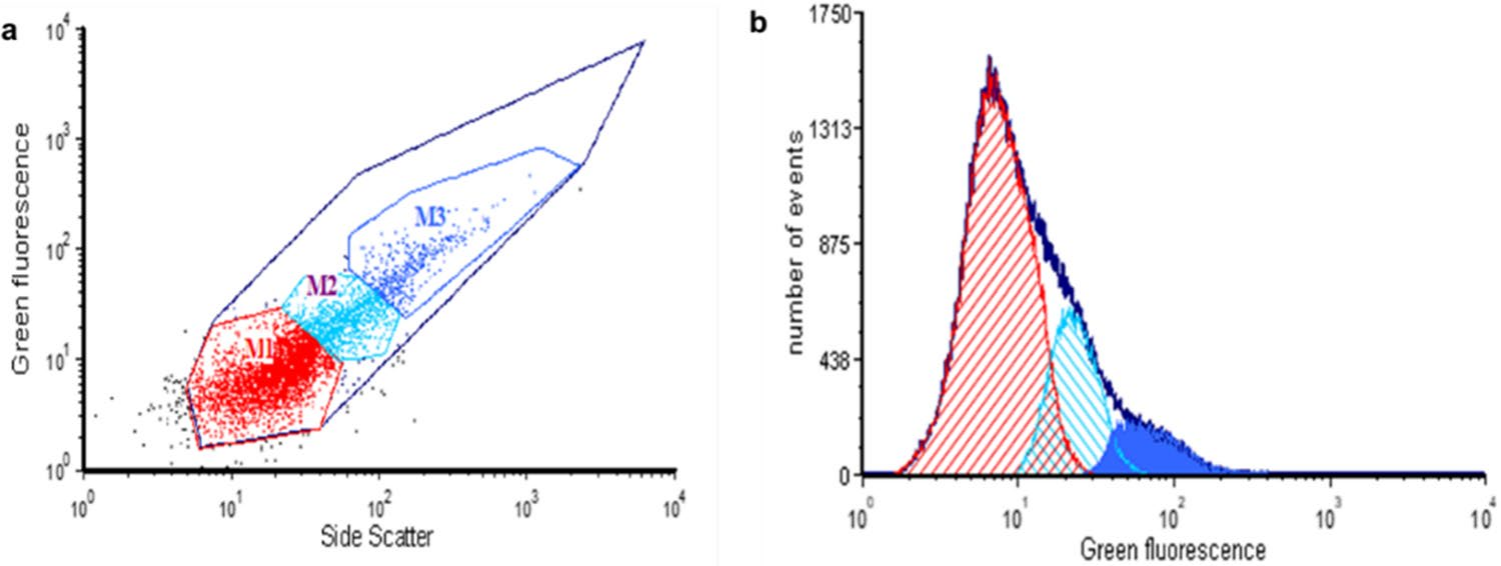
FACS analysis of progenitor cells. **a** Cytogram of Side Scatter (SSC, *x*-axis in the graph) and Green fluorescence (530/40[488], *y*-axis in the graph) shows fluorescent (dark blue line) of the progenitor, in which three main populations were discriminated based on fluorescence signal M1 red, M2 light blue, and M3 blue. **b** Histogram representing the fluorescence intensity of the three populations; the solid region indicates the population M3 which is the one with the highest green fluorescence signal. The graphs were drawn with FCS Express 6 flow v6.06.0033

**Fig. 2:**
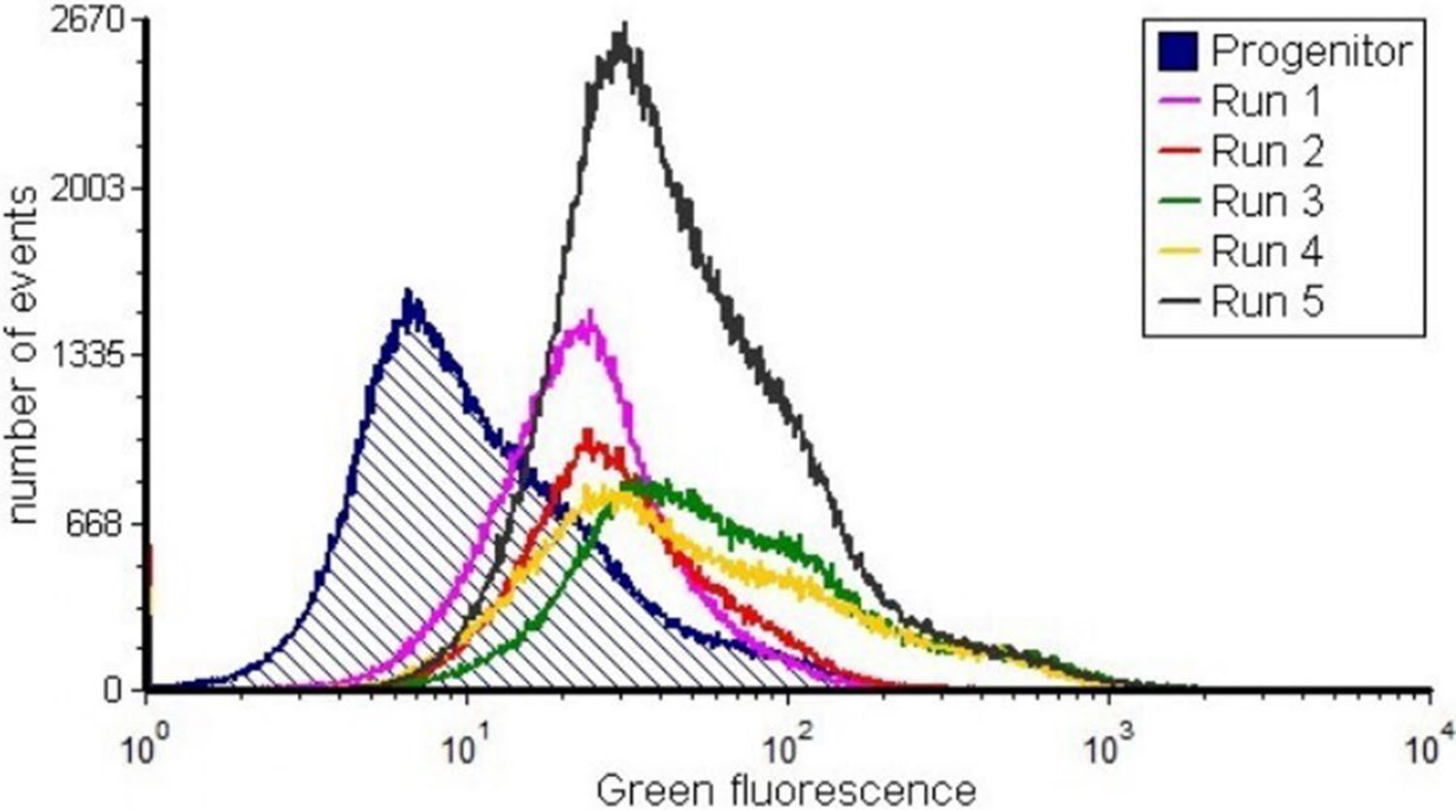
Histogram of FACS sorting. The random library was subjected to five runs of FACS. The dark blue line shows the progenitor. The first run, second run, third run, fourth run, and fifth run are represented by pink, red, green, yellow, and black curves, respectively. The maximum increase in Green fluorescence (530/40[488] on the *x*-axis was seen after the third sorting (green line). The graphs were drawn with FCS Express 6 flow v6.06.0033

The KrPL (pMAI79-*R9-gfp*) recombinant strain (from now on called “the progenitor”) was preliminarily analyzed by FACS. It was grown in the GG medium at 15 °C, and the expression was induced with 5 mM IPTG when the optical density of the sample ranged between 1 and 1.5. After 24 h from the induction, the cell culture was analyzed by FACS, and the Side Scatter (SSC) and Green fluorescence (530/40 nm) of each cell were correlated (Fig. 1a). The progenitor population could be divided into three populations (M1, M2, and M3) based on differences both in size signal and fluorescence intensity. The distribution of R9-GFP fluorescence among the cells (Fig. 1b) also confirmed that they could be divided into the main population (M1) and two subpopulations with higher fluorescence (M2–M3). Subsequently, KrPL cells harboring the randomly mutated psychrophilic plasmid library were subjected to FACS analysis, where the recombinant cells were sorted based on a combination of cell size (SSC) and their fluorescence as a proxy for their respective PCN (Fig. 2). A stock culture of recombinant KrPL cells (containing a number of clones representative of the whole library) was first propagated in the TYP medium at 15 °C, and then diluted in the GG medium. Finally, the R9-GFP recombinant production was induced by adding 5 mM IPTG for 24 h. To isolate the most fluorescent clones, the whole library was sorted by FACS. The collected subpopulation (called X population) was propagated in a small volume of the TYP medium at 4 °C; after 5 days, it was diluted in the GG medium at 15 °C and prepared for the round of sorting under the same conditions as described above. After the second sorting run, the subpopulation of the most fluorescent cells was collected, obtaining the Y population. Repeating this procedure, we collected the Z population after the third sorting run, the A population after the fourth one, and the B population after the fifth sorting run. As shown in Fig. 2, the fluorescent cells began to be enriched after the second run compared with the progenitor. The maximum increase in fluorescence was seen after the third run of sorting.

### Analysis of FACS-sorted psychrophilic populations and molecular characterization of some selected clones

Approximately 60–100 clones coming from each sorted population (X, Y, Z, A, B) were randomly selected, cultured in the selective GG medium at 15 °C in 12-well plates, IPTG-induced following the abovementioned production conditions, and subjected to spectrofluorimetric analysis. Figure S2 shows, as an example, the distribution of fluorescence intensities observed in the 60 clones from the screening of the Y population.

**Fig. 3:**
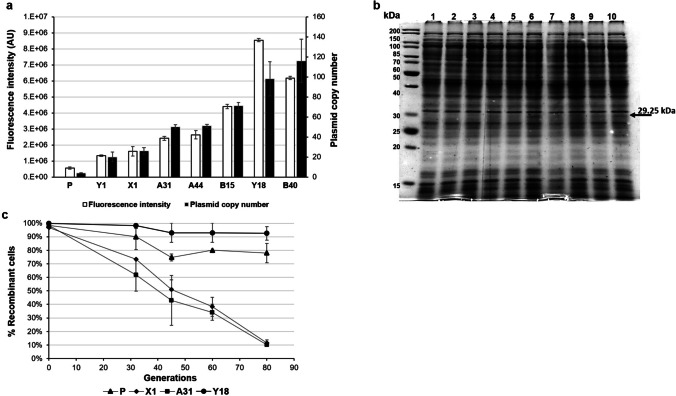
Characterization of the seven selected psychrophilic clones carrying mutant OriR sequences. **a** Analysis of the R9-GFP expression by spectrofluorimetry (open bars) and determination of plasmid copy number by qPCR (solid bars) of the progenitor (P) and selected clones (X1, Y1, A31, A44, B15, B40, Y18) are plotted on bar graph. The fluorescence of the progenitor and the clones indicated on the *x*-axis was monitored on induced cells (5 mM IPTG) after 24 h from induction. Fluorescence intensities are reported in arbitrary units (AU) as mean ± SD. **b** SDS-PAGE analysis of cell extracts of KrPL strains producing R9-GFP after 24 h from induction. lane 1, non-induced progenitor; lane 2, non-induced Y18; lane 3, induced progenitor; lane 4, induced X1; lane 5, induced Y1; lane 6, induced Y18; lane 7, induced A31; lane 8, induced A44; lane 9, induced B15; lane 10, induced B40. Black arrows on the right of the gel represent the expected molecular weights of the recombinant proteins. Black arrows inside the gel highlight the bands of the R9-gfp. **c** Plasmid stability assay. Plasmids pMAI79-R9-gfp (P, ▲), pX1-R9-gfp (♦), pA31-R9-gfp (■), pY18-R9-gfp (•) were separately propagated in KrPL for 80 generations without antibiotic selection. Plasmid stability was determined by replica plating onto selective media and presented as a percentage of cells that retain antibiotic resistance. Each experiment was carried out as biological duplicates and the error bars represent standard deviations

Once ruled out, the occurrence of any deleterious effects on recombinant cell growth possibly due to either replication or segregation instability of the high-copy number plasmids (Fig. S3), 7 clones (X1, Y1, Y18, A31, A44, B15, and B40) were further selected after the population’s analyses. Figure 3a (open bars in the graph) shows that all the selected mutants have a GFP-related fluorescence value higher than that of the progenitor, and the highest fluorescence intensity was recorded for the Y18 clone (8.55 × 10^6^ arbitrary units), which is approximately 15-fold higher than the one of the progenitor. Quantitative PCR (qPCR) was performed to determine the direct correlation between the higher fluorescence intensity and each plasmid copy numbers. The obtained results are reported in Fig. 3a (solid bars in the graph, and Table 1). Interestingly, all the selected clones contain recombinant plasmids maintained at higher PCN than the progenitor, with the highest PCN detected in the case of the B40 clone and evaluated as 116 ± 21.69 plasmid copies per cell, which is approximately 30-fold higher than that of the progenitor.

**Table 1:** Summary of nucleotide substitutions found in the selected OriR mutants. Nucleotide numbers are in agreement with the pMtBL sequence numbering (NZ_AJ224742.1)

Clone	PCN	Mutations							
X1	20 ± 4.99	T2399G							
Y1	26 ± 3.29	G2492T							
A44	50 ± 2.32	G2405A	A2472G						
A31	51 ± 1.54	T2051A	A2348G	G2452A	C2496T	T2563C	A2607C	A2698G	T2712C
B15	71 ± 3.45	T2446G	C2505A	A2608T	T2653A	T2660C	G2753T		
B40	98 ± 17.13	G2428A	G2506T	A2556T	T2569C	A2625G	G2684A		
Y18	116 ± 21.69	T2260C	C2491A	T2517A	T2544G	C2602A			

Finally, the production of R9-GFP was assessed in the total cellular extracts of IPTG-induced recombinant KrPL cells. Figure 3b shows a Coomassie-stained SDS-PAGE gel, in which the protein bands assigned to the overproduction of R9-GFP (29.25 kDa) are indicated by black arrows. Interestingly, a distinct extra-band is visible only in the induced cells containing the higher PCN mutant plasmids, i.e., plasmids pY18_79-*R9-gfp*, pB15_79-*R9-gfp*, and B40_79-*R9-gfp* (lanes 6, 9, and 10, respectively, in Fig. 3b). The lower production yield of the recombinant protein driven by the original plasmid and by the other mutants is consistent with the lower gene dosage of the recombinant cassette due to the lower PCN.

Since mutations introduced in the replication origin may affect the plasmid segregational stability, we compared the loss rate over 80 generations of three vectors (pX1_79-*R9-gfp*, pA31_79-*R9-gfp*, and pY18_79-*R9-gfp*) with that of the progenitor under non-selective conditions (Fig. 3c). These mutant vectors were selected because their PCN ranged from 20 (pX1_79-*R9-gfp*) to approximately 116 (pY18_79-*R9-gfp*)*.* Results shown in Fig. 3c indicate that the higher copy number plasmids (pY18_79-*R9-gfp*) have higher stability compared to the progenitor. The other two mutant plasmids, pX1_79-*R9-gfp* and pA31_79-*R9-gfp*, showed an average disappearance rate of resistant cells of 30% every 30 generations. Notably, vectors pX1_79-R9-gfp and pA31_79-R9-gfp exhibited stability of over 40% up to 45 generations, but this decreased to 10% after 80 generations.

### Sequence analysis of the isolated mutant plasmids and deletion mutations affecting the replication sequence

The nucleotide sequences of the OriR mutants in the seven selected clones (X1, Y1, Y18, A31, A44, B15, and B40) were determined and compared with the wild type from pMtBL. The summary of the detected substitutions is listed in Table 1. Interestingly, we found two clones (X1 and Y1) in which a single substitution was responsible for the increase in the copy number of approximately 20 (T2399G and G2492T, respectively).

**Fig. 4:**
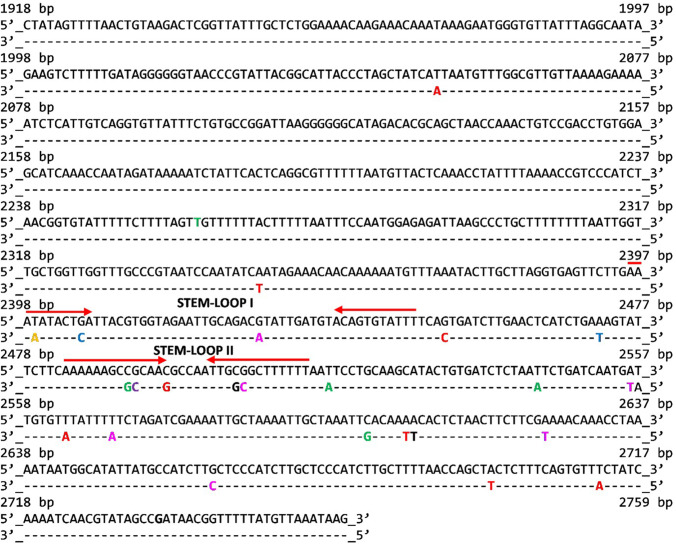
Localization of nucleotide substitutions in the selected mutants OriR sequences. The nucleotide substitution/s found in each mutant OriR is reported below the corresponding wild-type position using the following color code: yellow indicates substitution in clone X1; purple for clone Y1; green for Y18; red for A31; blue for A44; black for B15; pink for B40. The complementary sequences representing the stems of two putative stem-loop structures (STEM-LOOP I and STEM-LOOP II) are indicated with red arrows

Analysis of the sequencing data highlighted a nonhomogeneous distribution of mutations along the entire region (Fig. 4). In fact, all the mutations are located in the last four hundred nucleotides of the OriR sequence, except for one of the eight found in the A31 clone. DNA/RNA secondary structures can play a crucial role in regulating the plasmid copy number (Camps 2010) and point mutations in these structures may affect the affinity of the interaction and thus alter regulatory mechanisms (Camps 2010). Therefore, the Mfold WebServer tool was used to predict the DNA/RNA secondary structures that occur in the second half of the pMtBL OriR sequence. The software identified several putative secondary structures throughout the entire sequence, but our attention was focused on those in which the single nucleotide substitution found in the X1 and Y1 clones occurs. In both cases, the substitution affected the complementarity of the putative stem-forming sequences of STEM-LOOP I (position T2399G in the X1 clone) and STEM-LOOP II (position G2492T in the Y1 clone). To collect evidence on the potential destabilizing effect of the nucleotide substitutions on the suggested secondary structures, their minimum free energy value was computed and turned out to be higher than the value computed for the wild type pMtBL OriR (Table S2). Furthermore, many other nucleotide substitutions (found in the other selected clones) mapped in the same complementary sequences forming the stem of the two putative stem-loop structures. In all the cases, the mutations induced an increase in the calculated ΔG values of the corresponding secondary structures. To test whether the secondary structures contained herein are a necessary feature for the replication mechanism two OriR deletion mutants, i.e., devoid of the two putative stem-loop, was generated. The deleted origin turned out to be unable to support the plasmid replication in the Antarctic cells, suggesting that the deleted DNA region is essential for the mechanism of replication.

As for the almost “invariant” OriR sequence (from nucleotide 1918 to 2259), an in silico prediction of open reading frames (ORFs) distribution was performed. At least 13 putative ORFs were found that could potentially encode polypeptides between 10 and 66 amino acids. Unfortunately, a BLAST search revealed that none of them displays a worth mentioning homology with any other entry in publicly available databases. To understand whether this region is required for pMtBL replication, a pMAI79-*R9-gfp* derivative carrying an OriR mutant (devoid of the sequence from nucleotide 1918 to 2259) was constructed. Also, in this case, this specific deletion in pMtBL OriR resulted in a sequence unable to sustain the recombinant plasmid replication.

## Discussion

The production of recombinant proteins is essential in industrial biotechnology, and the efficacy of this process is influenced by several parameters such as host features, process temperature, promoter strength, and the number of copies of the expression vector used (Lozano Terol et al. 2019). This latter aspect has a strong impact on the productivity of the system as a high plasmid copy number (PCN) enables the achievement of the gene dosage required for efficient recombinant protein accumulation. In bacteria, large differences in the PCN are associated with different replication origins (Kim et al. 2020). For example, pMB1 is a medium-copy number episome (15–20 copies/cell) (Rossi et al. 1996), but some pMB1 derivatives, such as pUC, produce up to 700 copies/cell (Rossi et al. 1996). In the case of the plasmid pMtBL (Tutino et al. 2001), isolated from the Antarctic marine bacterium *Pseudoalteromonas haloplanktis* TAC125, the copy number is very low (Qi et al. 2019), and the PCN of pMtBL-derived vectors may limit the full exploitation of this bacterium as an unconventional host for recombinant protein production. Therefore, to improve the performance of the psychrophilic expression system, we decided to modify the origin of replication of pMtBL-based vectors to obtain a new generation of plasmids characterized by a higher copy number.

Since the replication mechanism of pMtBL is unknown, a random mutagenesis strategy appeared to be the most suitable approach to select more efficient origins of replication. A library of plasmids containing random mutations in OriR was constructed and analyzed in the KrPL strain. The collection of recombinant psychrophilic cells was then sorted by a FACS-based high-throughput screening method (HTS). Preliminary FACS analysis of the progenitor cells, i.e., KrPL cells carrying the wild-type pMAI79-*R9*-*gfp* plasmid, revealed a remarkable heterogeneity within this genetically homogeneous population. Similar heterogeneity has been previously observed in cells harboring low-copy number plasmids (Jahn et al. 2016). As plasmids follow a discrete distribution, a low mean PCN may also lead to higher cell-to-cell heterogeneity in PCN and gene expression. Kittleson and colleagues demonstrated a clear relationship between the PCN and the degree of heterogeneity in plasmid-encoded gene expression (Kittleson et al. 2011). Therefore, the observed progenitor heterogeneity was expected and prompted us to use a screening strategy that combined the FACS sorting with a subsequent screening step based on determining the fluorescence intensity of some isolated clones. By applying this approach several times, we isolated seven recombinant clones from different sorting steps characterized by psychrophilic plasmids maintained at different copy numbers, ranging from 20 to more than 110 (Fig. 3a). The ability to use genetic systems with different PCNs is advantageous under certain experimental conditions when tighter control of gene expression is required, and low-to-medium copy number plasmids may offer many more advantages compared with high copy number plasmids (Carrier et al. 1998). Furthermore, KrPL cells carrying the selected plasmids drive R9-GFP production to varying degrees, up to 15-fold higher than that observed in the progenitor (Fig. 3a), without showing any signal of metabolic stress (Fig. S3). Metabolic load generally correlates with plasmid copy number and is associated with cellular resource utilization for plasmid replication and recombinant protein production (Silva et al. 2012; Tan et al. 2020; Li and Rinas 2021). It is interesting to note that the recombinant production of human cyclin-dependent kinase-like 5 (*h*CDKL5) in *Ph*TAC125 (Fondi et al. 2021), obtained thanks to a vector containing the B40 OriR, resulted in a metabolic burden confirming that this effect is not related to the plasmid features but it is due to the recombinant protein characteristics.

Another critical aspect, associated with high copy number plasmids, is their intrinsic genetic instability. We examined the maintenance of the plasmid carrying the mutated OriR sequences over several generations. The plasmid stability assay (Fig. 3c) showed that the plasmid with a higher copy number (pY18_79-*R9-gfp*) had higher stability than the progenitor. In contrast, plasmids (pA31_79-*R9-gfp* and pX1_79-*R9-gfp*) with a medium-copy number showed lower stability, although approximately 50% of plasmid-containing cells are still present after 40 generations. The segregation of low-copy number plasmids is usually due to partition systems that actively distribute plasmid copies into the daughter cells. In the case of pMtBL, the occurrence of a potential partitioning *parABS* system was recently demonstrated (Qi et al. 2019). Encoded by a plasmid sequence outside the OriR region, this system cannot contribute to the segregation stability of OriR-derived plasmids in KrPL cells (Qi et al. 2019). In the absence of a partitioning system, successful plasmid segregation depends on the multi-copy state and the physical distribution of the plasmids inside the cell (Reyes-Lamothe et al. 2014; Wang et al. 2016). Therefore, higher stability of pY18_79-*R9-gfp* was expected while in the case of pA31_79-*R9-gfp* and pX1_79-*R9-gfp*, the lower stability compared to the progenitor might be related to the specific mutation(s) present in these plasmids and/or in the formations of plasmid multimers (Bedbrook and Ausubel 1976) (data not shown). Indeed, the formation of plasmid multimers may lead to a reduction in the number of heritable units during cell division, hence reducing the chance of successful segregation and consequently also the plasmid persistence over time (Summers 1998).

To obtain further information on the actual mechanism of pMtBL replication initiation and copy number regulation, the DNA sequence of the seven selected mutated OriRs was determined, and the identified base substitutions are listed in Table 1, where the experimental PCN values are also given. Interestingly, two of the seven analyzed clones are characterized by a single point mutation (X1 mutation in yellow, Y1 mutation in purple in Fig. 4), each occurring in the stem-forming sequence of a predicted stem-loop region. These results pointed the attention to these regions (indicated as inverted arrows in Fig. 4), and the observation that other clones, such as Y18 and B40, had mutations in these regions strengthened the hypothesis that the predicted DNA secondary structures might be involved in PCN control. Consistent with these considerations, our in silico analysis showed that the mutations that occurred destabilize the predicted secondary structures (Table S2).

Moreover, almost all the recorded mutations (except for one in the A31 clone) map in the last 350 nucleotides of OriR (region 2398–2712). The observed uneven distribution of the mutations along the OriR sequence from one side highlights the occurrence of a region of hot spot mutations, likely critical for the control of plasmid copy number, but also that the rest of the sequence does not allow the occurrence of any point mutation. Determining the functional role of this region (from 1918 to 2259 bp) and of the pMtBL replication mechanism is beyond the scope of this work, but what can be speculated is that the conservation of the original nucleotide sequence may underscore either the presence of small open reading frames (coding for small essential proteins) or the occurrence of functional sequences essential for the start or the progression of the plasmid replication. In light of this, the deletion of the first 342 bp of OriR abolished the ability of the remaining fragment to drive the psychrophilic plasmid replication. However, a transcriptomic analysis of the pMtBL plasmid showed that almost the whole plasmid is transcribed, with a higher than the average transcription involving the OriR region (1918–2759 bp) (Fig. S4). Interestingly, both the DNA strands are transcribed, although with different intensities, suggesting the occurrence of a replication mechanism based on complementary antisense RNA sequences, which may resemble what happens in the ColE1 plasmid. More focused work will be needed to describe the exact mechanism of pMtBL replication in detail.

The mutant OriR sequences studied in this work have several applications, fostering the development of the *Ph*TAC125 genetics and its industrial exploitation. Indeed, the availability of mutant OriR sequences, ensuring the establishment of higher copy number expression vectors, was essential to setting up the first conditional gene silencing system in a wild-type Antarctic bacterium (Lauro et al. 2022). The transcription efficiency obtained by combining the IPTG-inducible promoter (Colarusso et al. 2020) with the B40 PCN allowed establishing a sufficient paired termini antisense RNA concentration to switch off the expression of a medium-transcribed gene. This tool paves the way for the functional study of *Ph*TAC125 essential genes.

The implementation of the B40 in the pP79 vector represents a substantial improvement of the genetic system that makes now *Ph*TAC125 a real alternative host for the recombinant production of difficult-to-express proteins. Indeed, in the case of the full-length human protein* h*CDKL5 kinase, moving from pP79 vector to the improved system, the recombinant protein yield changed from few micrograms up to 5.2 mg (Fondi et al. 2021; Colarusso et al. 2022) per liter of culture. Beside the absolute production yield, the main biotechnological advantage of the Antarctic system consists in the quality of the human enzyme, which turned out to largely accumulate as full-length and catalytically active form, a result unmet in all the other microbial systems tested so far and that finally paves the way to the enzyme replacement therapeutic approach for this devastating genetic disease (Colarusso et al. 2022).

## Supplementary Information

Below is the link to the electronic supplementary material.

Supplementary file1 (PDF 507 KB)

## Data Availability

Correspondence and material requests should be addressed to MLT (tutino@unina.it) and MC (marzia.calvanese@unina.it).
