# Supplementary material for: Development of high-copy number plasmids in Pseudoalteromonas haloplanktis TAC125
Source: Appl Microbiol Biotechnol. 2023 Mar 13;107(7-8):2469–81. doi: 10.1007/s00253-023-12448-w (PMC10033558; doi:10.1007/s00253-023-12448-w)
Supplement: Supplementary file 1 — Supplementary file1 (PDF 507 KB) [file 253_2023_12448_MOESM1_ESM.pdf]

Development of high-copy number plasmids in *Pseudoalteromonas haloplanktis* TAC125

Marzia Calvanese[1], Cecilia Balestra[2], Andrea Colarusso[1,3], Concetta Lauro[1,3], Christopher Riccardi[4], Marco Fondi[4], Ermenegilda Parrilli[1], Maria Luisa Tutino[1,] *

[1] Department of Chemical Sciences, Federico II University of Naples, Complesso Universitario Monte S.-Angelo, via Cintia, 80126 Napoli Italia

[2] Istituto Nazionale di Oceanografia e di Geofisica Sperimentale, Oceanography Division – OGS, Trieste, Italy

[3] Istituto Nazionale Biostrutture e Biosistemi I.N.B.B, Viale Medaglie d'Oro, 305-00136, Roma, Italy

[4] Department of Biology, Via Madonna del Piano 6, 50018 Sesto Fiorentino, Florence, Italy.

*corresponding author: Maria Luisa Tutino, e-mail: tutino@unina.it , telephone +39081674317; fax +39081674313.

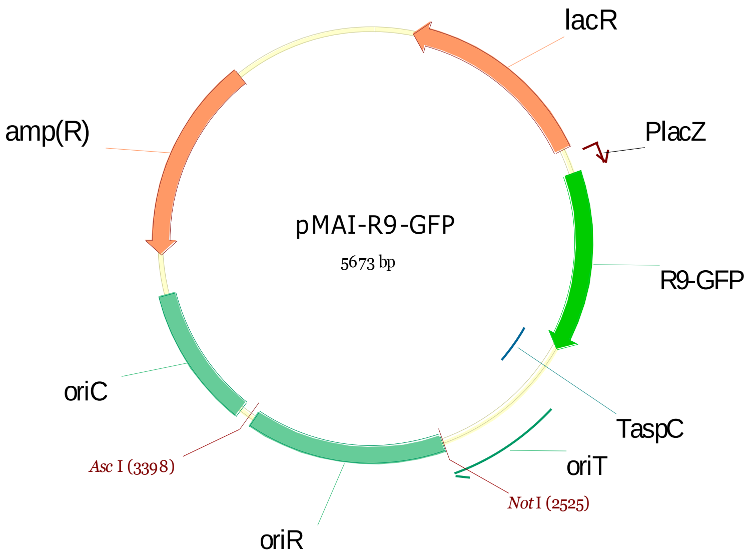

Fig. S1 Map of the pMAI79-*R9-gfp* plasmid. The two unique restriction sites (NotI and AscI) introduced upstream and downstream of the OriR sequence for the construction of the pMAI79-*R9-gfp* vector are highlighted in red. The map illustrates the genetic structure of the plasmid backbone including the pMtBL-derived replication origin for the maintenance in *Ph*TAC125 (OriR), the promoter (P*lacZ*), and the regulatory sequence (*PhlacR*) of lac operon recovered from the *Ph*TAE79, the transcriptional terminator of the *Ph*TAC125 *aspC* gene (T*asp*C), the conjugational DNA transfer origin (OriT), the β-lactamase encoding gene (*amp*^{R}) and the pUC18-derived replication origin for the propagation in *E. coli* (OriC).

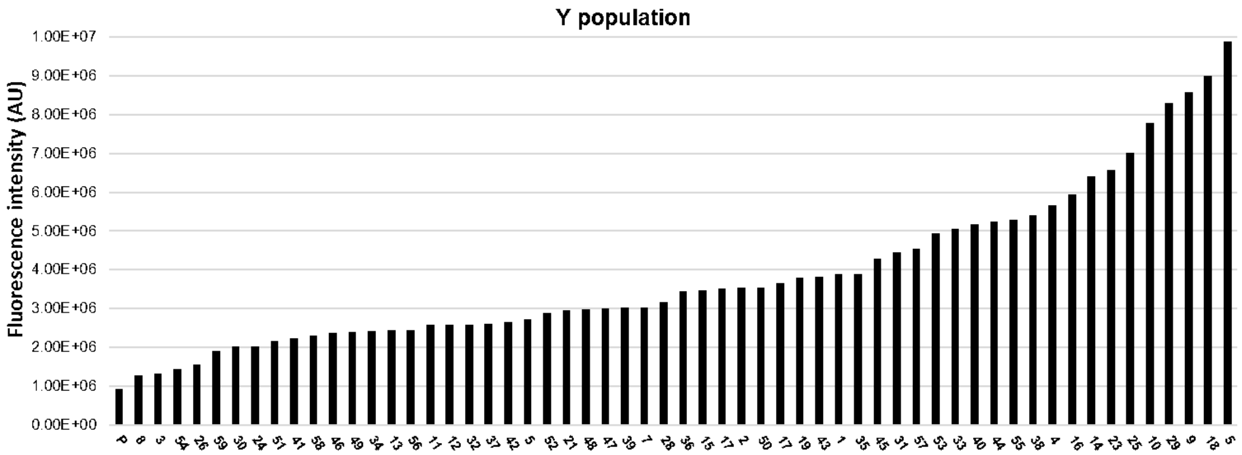

Fig. S2 Analysis of the R9-GFP expression by spectrofluorimetry. 60 clones of the Y population were randomly selected, cultivated in 96 deep well plates filled with the GG medium at 15°C, and harvested after 24 h from induction. The fluorescence of the progenitor (P) and the clones indicated on the x-axis are reported in arbitrary units (AU) as mean ± SD, n = 3.

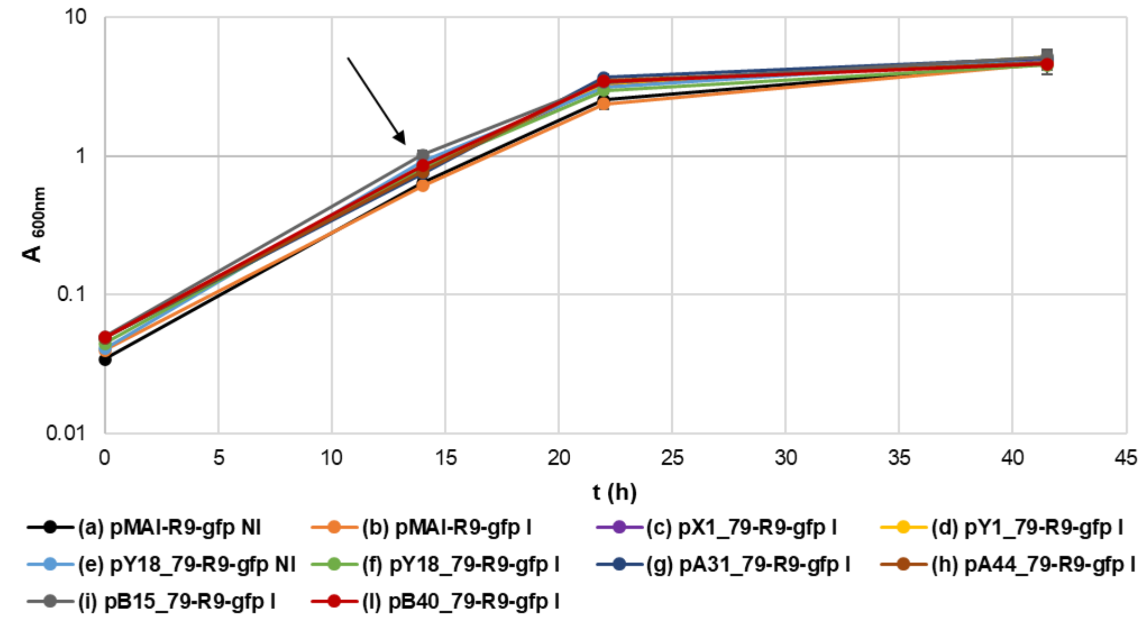

Fig. S3 Growth curves of KrPL harboring the original plasmid (pMAI-*R9-gfp*) and the high-copy plasmids. The recombinant cells were grown in the GG medium at 15 °C. Growth curves of non-induced cells harboring pMAI-*R9-gfp* (a) and pY18_79-*R9-gfp* (e). Growth curves of induced cells (5 mM IPTG) harboring pMAI-*R9-gfp* (b), pX1_79-*R9-gfp* (c), pY1_79-*R9-gfp* (d), pY18_79-*R9-gfp* (f), pA31_79-*R9-gfp* (g), pA44_79-*R9-gfp* (h), pB15_79-*R9-gfp* (i), and pB40_79-*R9-gfp* (l). The moment of the induction is represented with a black arrow.

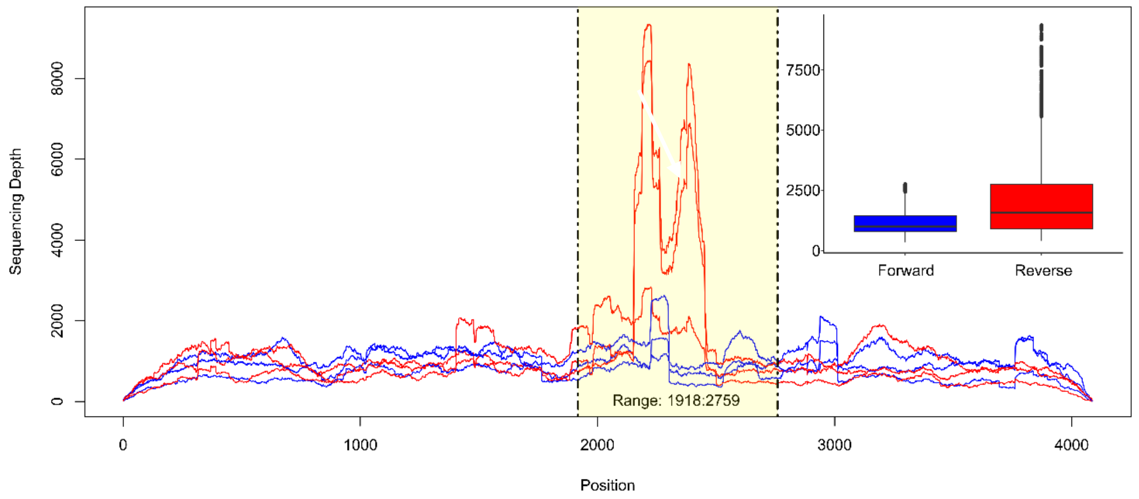

Fig. S4 Display of RNA-Seq data. Data from pMtBL transcriptome, the total coverage is displayed as a plot showing nucleotide-level sequence depth for the forward (blue) and reverse strand (red). The highlight focuses on OriR region, which stretches from nucleotides 1918 to 2759. Boxplot in the inset shows the quartiles of total forward (blue, mean: 1008.218, SD: 420.3342) and total reverse (red, mean: 2242.505, SD: 2123.815) strand sequencing depths for the highlighted region.

Table S1 List of bacterial strains, plasmids, and primers used in this study.

Strain	Relevant characteristics	References or Source
***E. coli* strains**		
DH5α	[supE44, ΔlacU169 (ϕ80 lacZΔM15) hsdR17, recA1, endA1, gyrA96, thi-1, relA1]	Lab stock
S17-1*(λpir)*	thi, pro, hsd(r− m+) recA::RP4-2-TCr::Mu Kmr::Tn7 Tpr Smr λpir	Tascon et al. 1993
XL10-Gold Ultracompetent Cells	TetR Δ(mcrA)183 Δ(mcrCB-hsdSMR-mrr)173 endA1 supE44 thi-1 recA1 gyrA96 relA1 lac Hte [F´ proAB lacIqZΔM15 Tn10 (TetR) Amy CamR]	Lab stock
***Ph*TAC125 strains**		
*Ph*TAC125 *wt*	Possesses two endogenous plasmids	Médigue et al. 2005 and CIP 108707
KrPL	*Ph*TAC125 cured strain without pMtBL plasmid	Lab stock (Unpublished data)
**Plasmid**		
pP79-*R9-gfp*	Expression vector with P*h*TAE79 *lacZ* regulative sequences producing enhanced GFP	Colarusso et al. 2020
pMAI79-*R9-gfp*	pP79-*R9-gfp* vector, introducing two unique restriction sites, *NotI* and *AscI*	This work
**Primer name**	**Sequence (5′ - 3′)**	
	**Multiple-site mutagenesis**	
Left OriR NotI Fw	CGGAAAAGATCCGTCGATTGCGGCCGCCTTATTTAACATAAAAAC	
Right OriR AscI Rv	TCAATTCATGTGAGCAAAAGGCGCGCCAAAGGCCAGGAACCGTAA	
	**Random mutagenesis**	
mut pP79-*R9-gfp* Fw	GAAAAGATCCGTCGATTGCGGCCGC	
mut pP79-*R9-gfp* Rv	GTTCCTGGCCTTTGGCGCGCCTTT	
	**Quantitative PCR**	
PSHAa2051 Fw	AACCGCACACAGACCCGAA	
PSHAa2051 Rv	AACGCACATTGGCATGACTGG	
R9-gfp Fw	GGAGAGGGTGAAGGTGATGCT	
R9-gfp Rv	GGTCAGAGTAGTGACAAGTGTTGG	
	**Deletions**	
OriR_BsiWI Fw	GGCCGTACGGAAGAATACTTTCAGATGAGTT	
OriR_BsiWI Rv	CGGCGTACGAATTCCTGCAAGCATACT	
OriR_NsiI Fw	GGCCGTACGGAAGAATACTTTCAGATGAGTT	
OriR_NsiI Rv	CGGCGTACGAATTCCTGCAAGCATACT	
OriR_NotI Fw	CTCGCAGAGCAGGATTCCCGTTGAG	
OriR_AscI Rv	CGCGGCGCGCCTGTTTTTACTTTTTAA	

Table S2 Free energy values (kcal/mol) of RNA and DNA secondary structures in the selected mutants compared to wild-type OriR were estimated with the Mfold tool

Sample	RNA structure		DNA structure	
	**Stem-loop I** Free energy minimum (kcal/mol)	**Stem-loop II** Free energy minimum (kcal/mol)	**Stem-loop I** Free energy minimum (kcal/mol)	**Stem-loop II** Free energy minimum (kcal/mol)
**wild-type**	-4.12	-17.45	-3.50	-14.67
**X1**	-3.81	/	-1.60	/
**Y1**	/	-11.66	/	-9.24
**Y18**	/	-11.47	/	-10.52
**A31**	/	-19.57	/	-16.05
**A44**	-2.57	/	-1.75	/
**B15**	-3.08	-11.77	-2.92	-10.40
**B40**	-2.69	-11.26	-2.07	-9.64
